# Successful implementation of parenting support at preschool: An evaluation of Triple P in Sweden

**DOI:** 10.1371/journal.pone.0265589

**Published:** 2022-04-13

**Authors:** Anton Dahlberg, Raziye Salari, Karin Fängström, Helena Fabian, Anna Sarkadi

**Affiliations:** Child Health and Parenting (CHAP), Department of Public Health and Caring Sciences, Uppsala University, Uppsala, Sweden; University of Wyoming College of Health Sciences, UNITED STATES

## Abstract

Although emotional and behavioural problems among young children are common and, if unaddressed, can lead to multi-facetted problems later in life, there is little research investigating the implementation of parenting programs that target these problems. In this study, the RE-AIM framework was used to evaluate the implementation of the Triple P parenting program in a preschool setting at a medium-sized municipality in Sweden. Reach increased over time, showing an overall increase in participating fathers and parents with lower education. Effectiveness outcomes showed an improvement in emotional and behavioural problems in children and less mental health-related symptoms and higher self-efficacy in parents. Adoption rate was 93.3%. To ensure staff “buy-in”, designated coordinators made changes in recruitment procedures, and provided supervision and training to all Triple P practitioners. Implementation adaptations were made, such as minor revisions of parenting strategies and other program content, as well as providing child care during seminars and groups, and setting up weekend-groups. Maintenance assessed through 12 month follow-up data suggested that several child and parent outcomes were maintained over time. Uppsala municipality continues to offer Triple P to parents. The reach, effectiveness, adoption, implementation and maintenance of the program were all satisfactory and demonstrated the suitability of delivering evidence-based parenting support using preschools as an arena.

## Introduction

### Emotional and behavioural problems in children

At preschool-age, two of the most common mental health problems are emotional and behavioural problems [1, 2]. This includes poor emotion regulation, anxiety or worries, moodiness, noncompliance, high activity level, poor impulse regulation, and aggression toward peers and/or adults [1, 3].

The reported prevalence of these problems varies heavily depending on source and methodology, but ranges between approximately 10 and 20% [4–6]. If unaddressed, these problems are generally stable over time, might also increase in severity [7, 8], and are associated with other mental health and social problems later in life [9, 10]. Thus, early interventions do not only have potential effects on current problems but might also reduce the risk of problems later in life.

Research from the past decades suggests that ineffective, coercive, harsh and inconsistent parenting contributes to preschool children’s disruptive behaviour and lack of self-regulatory skills [11–13], while proactive and responsive parenting is associated with lower levels of externalising behaviour problems and conduct problems [14]. Additionally, this relation seems to be bidirectional to some extent [15, 16], meaning that child behaviour problems and ineffective and harsh parenting could be affecting one another simultaneously. Parenting programs, or parent training, have been proven to be effective interventions aimed towards addressing child emotional and behavioural problems [17–19], as well as parent psychosocial health [20]. Parent training is associated with lessened child behaviour problems through an increase in child self-regulation skills [21].

### Triple P—Positive Parenting Program

Triple P is a parenting program developed for parents of children aged 2–16 [22]. The overall objectives of Triple P are to increase parents’ competence in building a positive parent–child relationship, promoting prosocial behaviour, and managing common emotional and behavioural problems as well as developmental issues in children; to reduce parents’ use of coercive and punitive methods of discipline; to improve parents’ communication about parenting issues; and to reduce parents’ stress associated with raising children [23]. The Triple P program emphasises the parents’ impact on healthy child development as well as emotional and behavioural problems by helping the parents identify what might cause them and setting goals for change [24]. Triple P is available at five different levels, going from light, universal interventions to more intense, indicated ones. At each level, program delivery is available in individual, group or self-directed formats [25]. In Sweden, Triple P is available in three formats with different intensity levels: seminar series (level 2, brief parenting advice), individual counselling (level 3, narrow focus parent skills training), and groups (level 4, broad focus parent skills training). These are described further in the methods section. Triple P has been evaluated in a large number of studies, with general evidence for positive effects on child behaviour problems, child prosocial behaviour, parenting behaviour, parental depressive symptoms, parental self-efficacy, and parental stress [26–28]. Implementation studies have shown that although the majority of providers trained to deliver Triple P use it regularly, a substantial number of providers either never use the program or use it scarcely [e.g., 29–32]. Lack of support and supervision has been identified as an important barrier to adoption and sustainability of Triple P, while the opportunity to consult with other Triple P practitioners has been reported as a vital facilitator. Those who use Triple P often report using it with good fidelity [29, 33] and program satisfaction is generally high among participating parents [33–36].

### Implementing parenting programs at the community level

Both Triple P in particular and interventions addressing child mental health in general, are recommended to be used as a blend of universal and targeted levels of intervention to optimise reach and effects of the program [25, 37]. For a parenting program to reach all or most parents, it needs to be available through services accessed by the vast majority of families. It also needs to be devoid of stigma, display minimal perceived barriers, address a perceived and relevant need, and induce a sense of self-efficacy [38].

Parents in Sweden are seeking out parent training for various reasons: curiosity, specific problems that they need help to tackle, or following recommendations from friends [39]. Parents who perceive their own parenting capacity as low and believe their children have mental health or behavioural problems are more interested in receiving parenting support [40]. Further, it has been stated [40] that individual or group formats have the potential of reaching families with more difficulties, while lighter formats such as seminars reach parents with higher education, hence using only the latter delivery format might increase inequalities.

Fathers are generally underrepresented in parenting programs [41, 42], even though research indicates that they have a substantial impact on both child development and family functioning [43]. Parenting programs therefore need to actively address the needs and interests of fathers to attract their participation [44]. Studies that examine both mothers’ and fathers’ participation in parenting programs show that different factors might be related to mothers’ versus fathers’ participation. For example, one study has shown that while mothers tend to be more prone to attend parenting programs when they report more child behaviour problems, fathers are more likely to attend when they report more child emotional problems [45]. One of the strategies to increase access to parenting programs in the Swedish national strategy for parenting support, is to reach parents at preschools [46]. Almost all children in Sweden aged 2–5 (93.8%) attend preschool [47], which makes it a suitable arena for recruiting families for general parenting support. In previous studies on preschool teachers with Triple P training in Sweden, they expressed several positive aspects of working with parenting support within their workplaces: the training provided them with tools and a set of strategies to help and support parents; the program interventions focused on helping parents to find their own solutions to family-related problems; and the training and program delivery increased practitioners’ confidence in their role as preschool teachers, both regarding teacher–child interactions and in communicating with parents [48]. Although preschools are suitable for parenting programs and preschool teachers are well equipped as practitioners, there are very few studies where this arena has been utilised when implementing parenting programs [49, 50].

The present study describes the implementation of the multilevel Triple P parenting program using preschools as an arena in a medium-sized Swedish municipality (~230,000 inhabitants), during 2009–2018. The implementation was part of a national effort to increase parents’ access to parenting support and the project was a joint enterprise between the municipality and university researchers.

Successful implementation of research-supported interventions within the child welfare system is related to several common factors: funding and collaboration with external stakeholders (outside of an organisation); leadership support, staff burden, and agency culture (inside of an organisation); worker “buy in” and client resistance (individuals involved); program fit and intervention clarity (characteristics of intervention); providing appropriate training, supporting staff competency and having an implementation team supporting the implementation process (implementation process quality) [51]. Although preschools are not part of the child welfare system, the above-mentioned factors can be helpful in understanding the implementation of research-supported interventions in this setting as well, as they reside within the public sector and thus share a similar organisational structure and are financed through public money. To plan, implement and evaluate the introduction of Triple P, the RE-AIM planning and evaluation framework was used [52–54]. RE-AIM is one of well over 100 frameworks used (primarily) within dissemination and implementation research [55], and offers a structured way of employing, describing and analysing implementation processes at five key dimensions [56]. These dimensions are *reach*, *effectiveness*, *adoption*, *implementation*, and *maintenance* which are described in the methods section.

## Aim

The overarching aim of the study at hand was to assess the implementation of the Triple P multilevel parenting program in a preschool context, delivered by preschool teachers.

Being informed by the RE-AIM framework, this study set out to answer the following research questions:

- How many parents were reached by the parenting program and what were their characteristics in regard to SES and other demographic variables? (Reach)
- What outcome can be seen in terms of changes in child behaviour, parenting adjustment and parent mental health? (Effectiveness)
- How many preschools were participating in the implementation of Triple P within the municipality and what were their characteristics? (Adoption)
- Do the trained practitioners continue delivering seminars and groups? (Adoption)
- What adaptations were made to implement the intervention during the course of the study? (Implementation)
- Were any changes in child or parent outcomes sustained over time? (Maintenance)
- Are there any traceable long-term adaptations at setting level? (Maintenance)
- Does the intervention become part of routine organisational practices over time? (Maintenance)

## Materials and methods

### Procedure

There were two waves of data collection. During the first wave, 2009–2013, initial implementation concerns were addressed, informing adaptation of the program and staff-level composition. All other data for this study were collected during the second wave, 2014–2018. Parents were invited to participate in the program through flyers and posters with information about the program. There were no stated exclusion criteria for participating in the parenting program in the flyers or posters. However, since the program focus was on preschool children and their parents, recruitment took place mainly at preschools and child health clinics. There was also a webpage dedicated to the program, consisting of information about the available levels of Triple P in the municipality and dates for upcoming seminars and groups. Therefore, parents with preschool-aged children were most likely to be exposed to the program information.

#### Intervention

Triple P was offered at three different levels in the municipality: seminars (level 2), individual counselling (level 3), and group (level 4). As there were only 31 individual counselling series during the entire study period, and because of too little collected data from the parents, only the seminars and group levels were assessed in this study.

The Triple P seminar series consists of three stand-alone seminars each lasting two hours, where parents can attend in the order and as many as they see fit. The seminar topics are *The power of positive parenting*; *Raising confident*, *competent children*; and *Raising resilient children* [23]. Triple P group includes eight sessions in total. The first four are two-hour-sessions covering different aspects of parenting, such as information on child development, specific parenting skills and making a personal plan for change. Sessions 5 to 7 consists of 20–40 minute, individual phone calls to all parents, where individually tailored plans and parenting techniques are set up and evaluated together with the practitioner. In the final session, the program is summarised and evaluated, and parents are encouraged to make individual plans for sustaining the strategies and insights they have gained during the course of the program [23].

#### Evaluation

There was no need for registration to attend Triple P seminars, hence parents could show up to each seminar without prior notice. At the end of each seminar, every parent was asked to fill out an evaluation form containing questions concerning the quality and content of the seminar, as well as basic demographic data about the parent. This form could be used for evaluating program satisfaction, but not program effectiveness. For Triple P group, parents registered by contacting the Triple P coordinators via email. The coordinators then provided information about Triple P and the study at hand. Information letters were sent to the parents via mail one to two weeks prior to program start, containing information about the study, request for informed consent, and questionnaires for pre-measures. Parents were asked to complete the questionaries, sign the consent form indicating whether or not they were interested in participating in the study, and bring them to the first group session. Written informed consent was obtained from all participants included in the study. Questionnaires were also distributed during the last session of each group, enabling pre–post analyses. Further, at 12 months after completing the Triple P group, parents received a third set of questionnaires for follow-up analyses. Additionally, the practitioners kept an attendance list with the number of parents participating at each seminar and group session. No commission or payment was offered for study participation. The study was approved by the Regional Ethical Review Board in Uppsala, Sweden (*Dnr* 2009/161 and 2012/437).

### RE-AIM dimensions

#### Reach

Reach is defined as the proportion and representativeness of the individuals who participated in Triple P at either seminars or in groups. This was assessed through describing the characteristics of participating parents and children in relation to basic demographics of the general population. Data from the general population were retrieved through Statistics Sweden (SCB.se). For both groups and seminars, the participant rates were investigated, by describing parents attending one versus several seminars and those attending some group sessions versus all group sessions. Reach was also assessed over time, to detect changes during the study period. The data from the Triple P seminars were collected at parent-level, and hence the exact number of children was not assessable. However, as an orientation aid, the approximate number of preschool-aged children in the municipality was 11,750 during the second wave of data collection.

#### Effectiveness

Effectiveness is defined as the degree to which the level of primary outcomes of the Triple P group participants is changed. We assessed this through changes in parent and child outcomes using a pre–post design. Further, parents were asked to evaluate the seminars series after participation. Instruments to measure outcomes and evaluate the program are described under the heading “Instruments” further below.

#### Adoption

Adoption is defined as the number, proportion and representativeness of the settings (preschools) and staff using Triple P. To evaluate adoption at the setting level, the adoption rate (participating versus non-participating preschools) was assessed, as well as the characteristics of the participating sites in relation to the *Socioeconomic Structure Compensation* index. The Socioeconomic Structure Compensation index is an index used by some larger Swedish municipalities to guide resource allocation to preschools and schools, and is comprised by the socioeconomic composition of the individual children or pupils at each preschool or school [57].

In a report preceding the study at hand, adoption at staff level was assessed through interviewing staff. The report concluded that preschool teachers were positive towards implementing parenting support at preschools, and that they saw the program as providing useful tools and techniques for promoting positive child development. Further, assessment of staff “buy-in” indicated positive aspects of the program for the staff and its usefulness within the preschool staff´s own professional work [see 48]. In the present study, staff level adoption was assessed through describing the recruitment process and staff adoption rate. The practitioners were also interviewed to assess possible hindrances to the delivery of Triple P at staff level. Additionally, changes in the recruitment process were delineated. Successful adoption at staff level was considered if at least 80% of the trained staff used the program at the end of the second wave.

#### Implementation

Implementation is defined as the fidelity to the Triple P functions and components, including delivery as intended and intervention costs. This was assessed through adaptations made during the study, training of staff, cost of intervention, and consistency of implementation across staff. The Triple P coordinators also interviewed parents who had attended the program concerning their experiences as participants and asking for feedback on what enables or disables participation, from the parents’ perspective. Successful implementation was defined as fruitful adaptations and implementations of the program that managed to fit the needs of practitioners and parents throughout the study period.

#### Maintenance

Maintenance is defined as sustained changes in outcomes at individual level and sustained use of Triple P at the organisational level. Data from 12 months follow-up questionnaires were used to measure whether potential changes after group-attendance lasted over time. Further long-term attrition was analysed to assess any differences between parents answering the follow-up questionnaires and those who did not reply. At the organisational level, maintenance was measured through assessing participation at seminars and groups over time. Additionally, long-term adaptions of the program were described, as well as its compatibility with the municipal policy for sustainable development. Maintenance was deemed successful if potential changes in parent and child outcomes where traceable over time, and if the Triple P seminars and group participation were undiminished across the study period.

### Instruments

Instruments to measure changes before and after group attendance, as well as evaluation forms for seminars, are described below. As the group questionnaire package contained several questionnaires, some specific questionnaire subscales were omitted from the questionnaire packages in favour of focusing on the studied outcomes and keeping the questionnaire packages as short as possible. Omitted subscales are described where applicable.

#### Child emotional and behavioural problems

To measure children’s behavioural and emotional problems, the Strengths and Difficulties Questionnaire (SDQ) and Eyberg Child Behaviour Inventory (ECBI) were used. The SDQ is a 25-item questionnaire, designed to assess positive and negative behaviours in children, through parents’ ratings of difficult as well as prosocial behaviours in their child [58]. Along with a total score of the child’s difficulties, five subscales measure conduct problems, emotional problems, hyperactivity, peer problems, and prosocial behaviour. Scores are computed by summing the scores for each item of the subscales, which are measured on a 3-point Likert-scale. The Swedish SDQ for preschool children has shown good construct validity, concurrent validity and internal consistency [59–61]. For this study, we strived to use age and gender specific norms from Swedish populations. This, however, was not entirely possible. The SDQ was scored based on age and gender specific norms for children aged 3–5 [62]; for 2-year-olds, general (non-age and non-gender specific) Swedish norms from children aged 1–5 were used [63]; for children age 6 and above, general Swedish norms for children aged 6–10 were used [61].

The ECBI is a 36-item rating scale measuring disruptive behaviour problems in children, assessed through an intensity scale and a problem scale, measuring frequency of a behaviour and whether the parent perceives the behaviour as a problem [64]. The Swedish version of ECBI has showed good internal consistency, convergent, discriminative and construct validity [65].

#### Parents’ adjustment

Parents’ mental health was assessed through the General Health Questionnaire (GHQ-12) and the Depression Anxiety Stress Scale (DASS). The GHQ-12 is a shortened version of the 60 item version of the GHQ, an instrument primarily measuring depressive symptoms but also anxiety [66, 67]. The Swedish version of the GHQ-12 has been validated and normed recently, indicating good psychometric properties, such as discriminant validity and internal consistency [68, 69]. The DASS measures depression, anxiety and stress symptoms [70], with indications of good convergent and discriminant validity [71]. The shorter 21-item questionnaire was used in this study which has been validated previously, both internationally and in a Swedish context [72–74]. In the current study, only the Depression and Stress subscales were administered.

#### Parenting

The Parenting Scale (PS) was used to assess dysfunctional discipline styles [75]. It comprises three subscales, measuring laxness (permissive discipline), over-reactivity (displays of anger and irritability, and authoritarian discipline), and verbosity (repeated use of verbal means of addressing misbehaviour). Since previous studies of the validity of the PS suggest the omission of the Verbosity subscale [e.g. 76–78], this study included only the Laxness and Over-reactivity subscales. The Swedish PS has been shown to have good internal consistency, test-retest reliability and predictive validity [77]. To assess self-efficacy, the Parenting Sense of Competence Scale (PSOC) was used [79]. PSOC has proven to have good construct validity and reliability [80, 81]. The questionnaire measures both parent satisfaction and self-efficacy. However, since self-efficacy was mainly of interest in this study, only the Efficacy scale was administered.

#### Parents’ relationship quality

To assess conflict between cohabiting parents, we used the Parent Problem Checklist (PPC), which measures inter-parental conflict in child-rearing matters and displays good overall reliability and validity [82, 83]. For measuring the relationship quality of co-habiting parents, the short version of the Dyadic Adjustment Scale, DAS-4 was used. The questionnaire contains four questions, measuring relationship satisfaction, with studies indicating good construct and predictive validity [84].

#### Program evaluation questions

Program evaluation questions were collected at the end of each Triple P seminar. There were eight questions in total, scored on a 7-point Likert scale, (see Fig 4 and S1 Appendix for the list of items). For the sake of this study, the variables were dichotomised into scores at 6–7 and scores below 6 respectively due to generally high ratings.

### Statistical analysis

All analyses were carried out using R 4.0.3 [85] and SPSS 26. T-tests were used to assess changes in child and parental outcomes after participating in Triple P group and at one year follow-up. To avoid type-I errors, Benjamini-Hochberg [86] correction of *p*-values was applied with a false discovery rate set to 5%. Further, Cohen’s *d* analyses were carried out for all significant results of the t-tests, to assess the magnitude of change after Triple P group participation and at 12 months follow-up.

To assess potential differences in change over time between participants with data from all three timepoints and participants with only pre and post measures, one-way MANOVAs were conducted for parents’ and children’s outcome variables. There were no significant differences in outcome scores between the two groups, neither for parents (F(2, 118) = 17.314, *p* = .839; Wilk’s Λ = .963) nor for children (F(2, 149) = .474, *p* = .701; Wilk’s Λ = .990). Further, chi squared analyses were performed on parents’ background variables regarding country of origin (foreign born), education level (university education), and marital status (single) between the two groups. No significant differences were found for parent country of origin (χ^2^ (1, 168) = .018, *p* = .894), education level (χ^2^ (1, 165) = .352, *p* = .553) or marital status (χ^2^ (1, 168) = .515, *p* = .473). This suggested that 12 months follow-up analyses could be generalised to the full sample.

## Results

Out of 2,275 seminar attendees, 2,021 anonymous seminar evaluation questionnaires were collected during the second wave. Of these questionnaires, 50 were excluded due to missing informed consent by the parent, leaving 1,971 questionnaires for analysing program reach and program satisfaction. The gender distribution of the children was even, with parents seeking parenting information for 55.5% boys and 44.5% girls.

**Fig 1:**
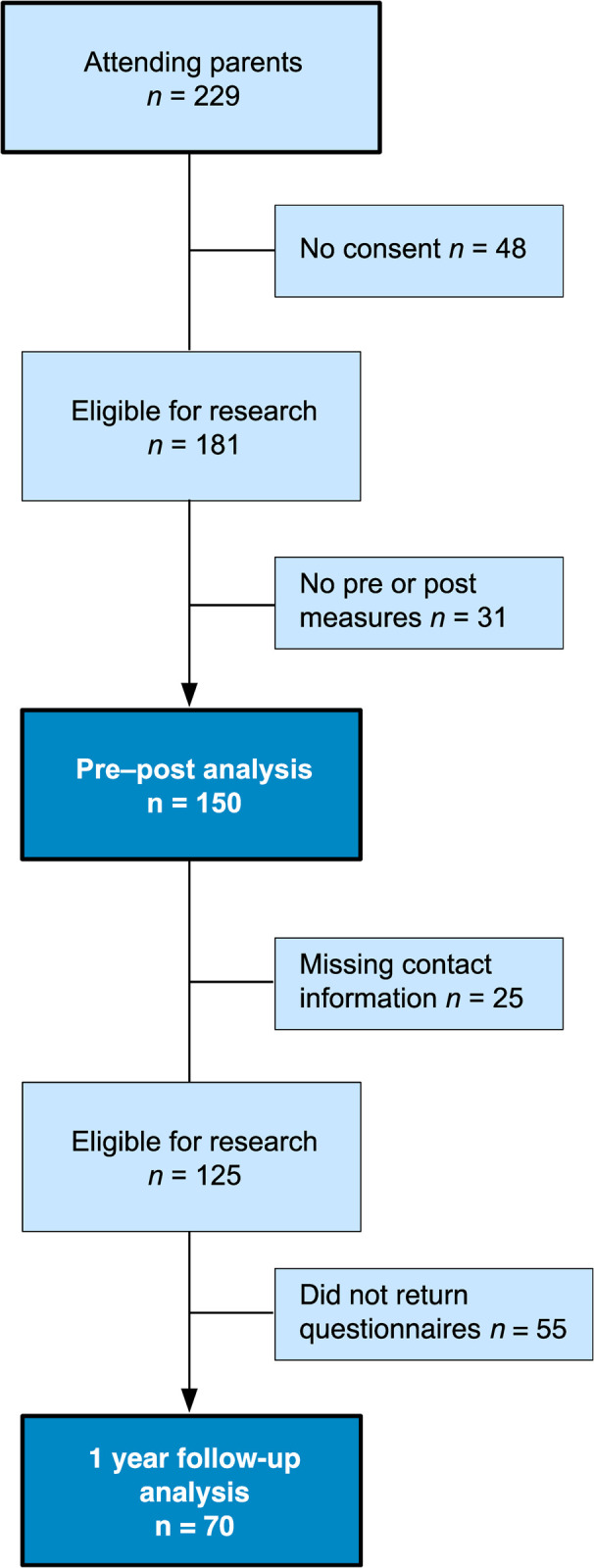
Flowchart of data exclusion for Triple P group.

Please see Fig 1 for a flowchart of the Triple P group data collection during the second wave. A total of 150 parents of 112 children with ratings from pre and post group attendance were included in the analyses and were collected during the second wave. All pre- and post-data collection was amassed on site, by the Triple P practitioners. For analysis of child outcomes, one questionnaire was selected for children with ratings from both parents. Hence, if a child was rated by both parents, the questionnaire with complete data was selected (*n* = 7). If both questionnaires were complete, one rating was randomly selected (*n* = 31). Follow-up questionnaires were sent out to all participating parents via mail if they had provided contact information (*n* = 125). Of these, 70 parents of 48 children filled out and returned the questionnaires. The gender distribution of the children in Triple P group was more uneven compared with seminars, with boys making up circa 1/3 of the sample.

### Reach

Age and basic demographic variables on parents and children for the entire study period are displayed separately for seminars and groups in Table 1. The overall proportion of preschool-aged children was 94.2% for seminars and 96.4% for groups. Single parent participants in both seminars and groups were fewer than in the general population. Foreign born parents were fewer than in the general population for both seminars and group formats. The proportion of unemployed parents was lower than in the general population for both seminars and group formats, with seminars being closer to the general population (Table 1).

**Table 1:** Age, gender distributions and basic demographic characteristics of children and parents during 2015–2019.

		Seminars	Groups	General municipal population
		*n* = 1,971	Children *n* = 112	
			Parents *n* = 150	
Child gender				
	Boys (%)	55.5%	68.5%	
	Girls (%)	44.5%	31.5%	
Child mean age (*SD*)		3.7 (2.0)	3.6 (1.5)	
Child age range		0–12	1–8	
Relation to child				
	Fathers	29.9%	40.0%	
	Mothers	70.1%	60.0%	
	Other	1.9%	0.0%	
Parent mean age (*SD*)		36.1 (5.8)	37.2 (4.8)	
Parent age range		20–60	26–48	
Highest education				
	Not finished primary school	0.3%	0.0%	2.6%
	Grade school	1.5%	3.2%	5.6%
	High school	27.4%	30.1%	28.6%
	University < 3 years	6.7%	6.2%	13.2%
	University ≥ 3 years	54.4%	50.5%	42.3%
	Graduate degree	9.7%	10.0%	7.8%
Parent born in Sweden				
	Yes	81.3%	80.4%	73.5%
	No	18.7%	19.6%	26.5%
Employment status				
	Employed (full- or part-time)	94.3%	96.6%	96.6%
	Unemployed	5.7%	3.4%	6.8%
Marital status				
	Married or partnered	91.5%	92.8%	86.7%
	Single	8.0%	7.2%	13.3%
	Other	0.5%	0.0%	0.0%

The proportion of parents attending Triple P group with high school education or lower was only 11.1% *during the first two study years*, and the proportion of foreign born parents amounted to 16.7% (not shown in the table). To address the lopsided distribution of foreign born parents and parents with lower education level, the Triple P coordinators intensified recruitment efforts in areas of the municipality with higher rates of foreign born parents, as well as in rural areas, where the overall education level of the population is generally lower. This began in 2017 and continued throughout the study period. Following these efforts, in 2018, the number of parents with lower education and foreign born parents increased to 30.6% and 25.0%, respectively. To attract parents from minority groups, the team liaised with local opinion leaders who helped facilitate recruitment.

In the group intervention, 49.1% of the children scored above cut-off on the SDQ, and 28.2% on the ECBI Intensity scale. Parents’ scores on the GHQ were high, with 43.2% scoring above cut-off. This indicated that the sample consisted of children with a higher degree of emotional and/or behavioural problems and parents with a poorer general mental health compared with the general population.

**Fig 2:**
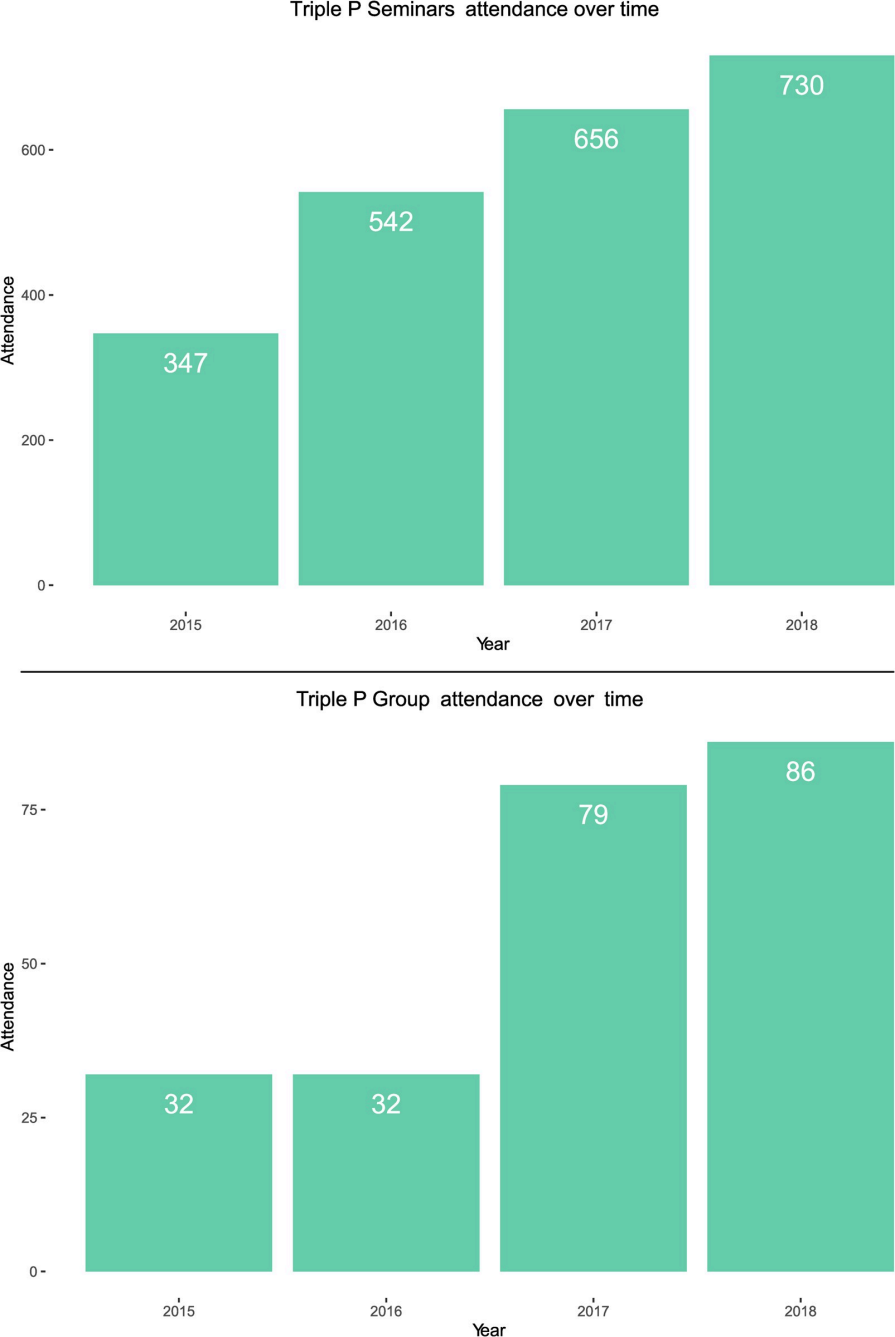
Attendance over time at Triple P seminars and groups.

**Fig 3:**
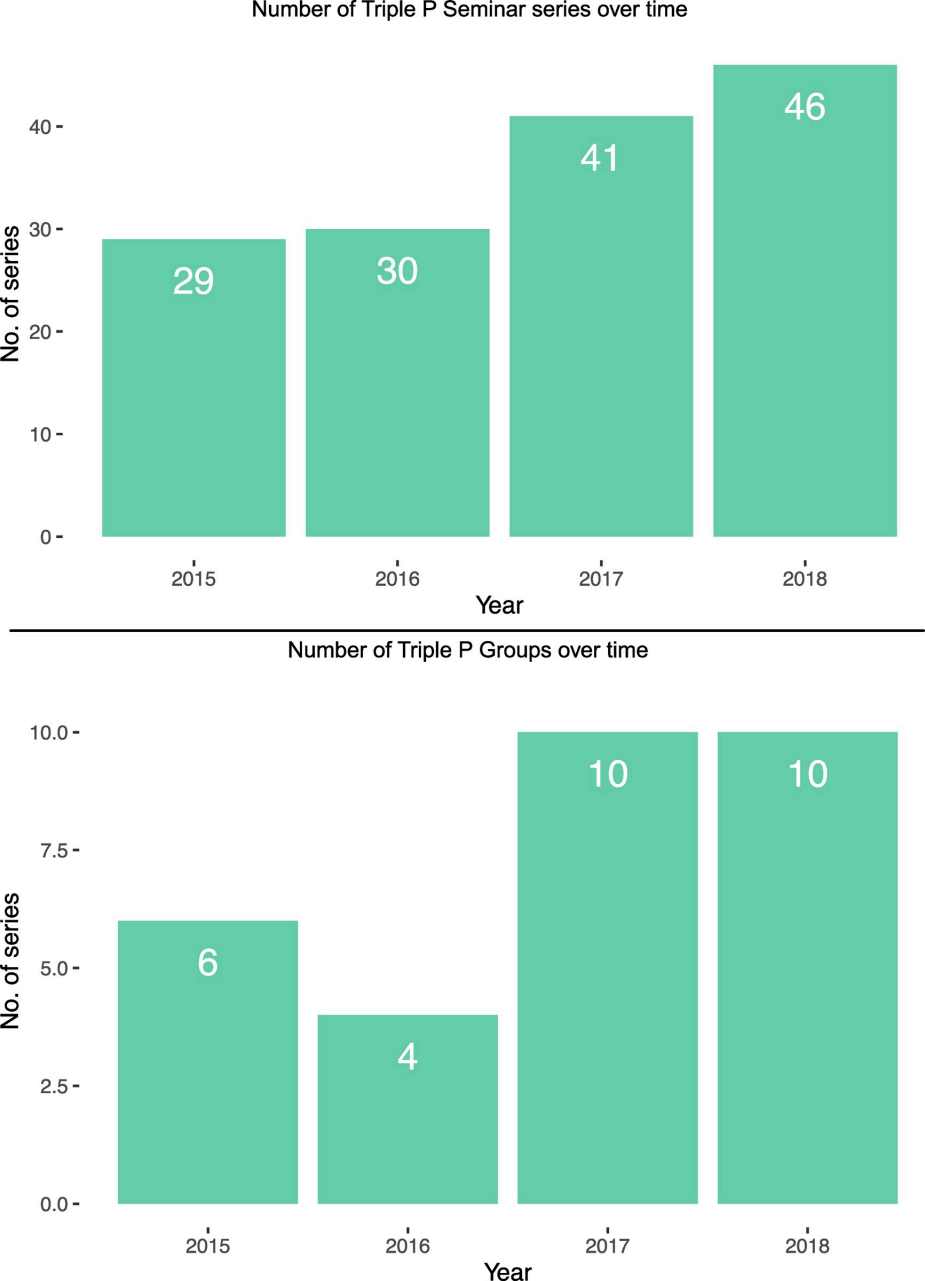
The number of seminar series and groups held over time.

Attendance increased over time for both seminars and groups, illustrated in Fig 2. Group attendants generally adhered to the program’s full set of sessions. Out of the 150 parents, participation records were available for 105 of them, which showed a 92.4% complete attendance. Attendance at seminars was evenly distributed across the series: 37.3% at seminar 1, 34.4% at seminar 2, and 28.4% at seminar 3. Parents attended an average of 1.5 seminars, with 63.1% attending one seminar, 23.2% attending two seminars, and 13.0% attending three seminars. The remaining 0.7% attended four to seven seminars. The coordinators and practitioners worked actively to attract more parents over time, which was reflected in the number of seminar series and groups offered every year. As can be seen from Fig 3, the number of seminar series and groups held increased over time.

### Effectiveness

#### Seminars program evaluation

**Fig 4:**
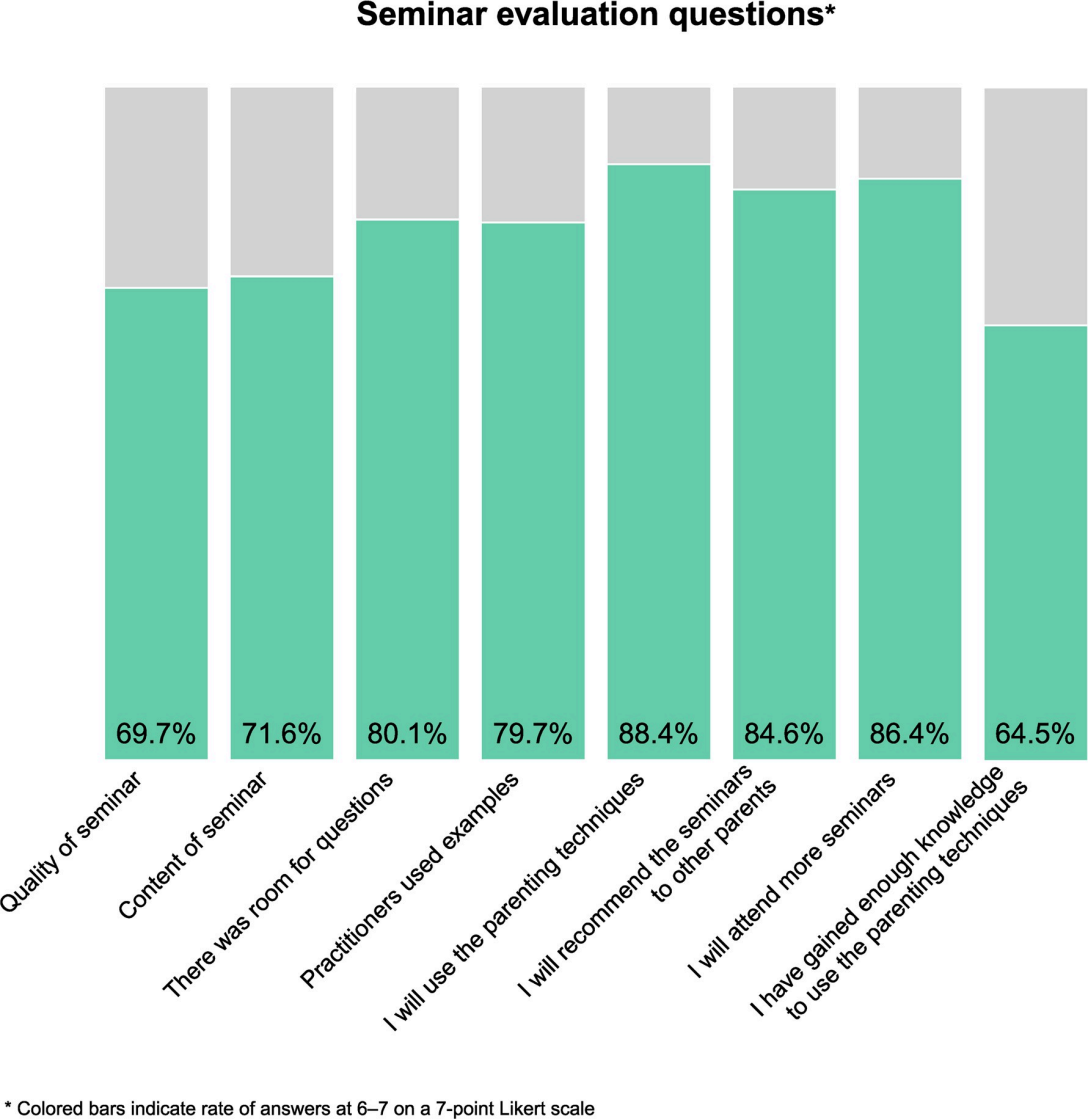
Percentage stacked bar charts of evaluative questions from seminars.

Seminar attendants scored the quality of the program as very high overall, as well as the likelihood that they would use the taught parenting techniques in their own parenting. Fig 4 displays all evaluation questions, with percentages of ratings at 6 or 7 on the 1-to-7 scale. As can be seen, parents were in general very pleased with the seminars and gave high ratings of every evaluative question. An additional question, “I would like to participate in a more intense level of Triple P” was asked in connection with the evaluation questions. With 42.9% of parents’ ratings at 6 or 7, this indicates an interest in parenting support at different intensity levels.

#### Groups pre–post analyses

Results from pre–post analyses of child and parent outcomes for Triple P group are presented in Table 2. Significant differences after participation in group Triple P were found for SDQ Total score and the SDQ subscales Conduct Problems, Hyperactivity and Prosocial Behaviour. Further, significant differences were found for the ECBI Intensity and Problem scales. No significant changes were found for the SDQ subscale Peer Problems. The results imply a positive change in behaviour problems overall, as well as a specific change concerning reduced conduct problems and an increase in prosocial behaviour.

**Table 2:** T-tests for Triple P group pre–post assessment.

		Pre		Post					* *
*Child outcomes* (*n* = 112)*		Mean	*SD*	Mean	*SD*	*t*	df	*p* †	*Cohen’s d*
SDQ Total		10.5	4.8	8.9	4.3	5.836	111	< .001	.55
	SDQ Emotional Symptoms	1.8	1.7	1.7	1.6	2.621	111	.011	.25
	SDQ Conduct Problems	3.2	1.8	2.6	1.6	5.441	111	< .001	.51
	SDQ Hyperactivity	4.0	2.4	3.6	2.3	2.762	111	.009	.26
	SDQ Peer Problems	1.4	1.6	1.2	1.4	1.725	111	n.s.	–
	SDQ Prosocial	6.8	2.1	7.3	2.0	-3.315	111	.002	.31
ECBI Intensity		121.2	25.7	106.9	23.1	9.042	111	< .001	.85
ECBI Problems		11.9	6.4	6.7	5.5	9.163	102	< .001	.90
*Parent outcomes* (*n* = 150)*									
GHQ-12		11.8	5.4	9.5	4.8	5.632	148	< .001	.46
PS Laxness		3.1	0.7	2.9	0.7	5.878	144	< .001	.49
PS Over-reactivity		3.3	0.8	2.8	0.8	8.643	145	< .001	.72
PSOC Efficacy		27.3	5.3	30.6	4.8	-9.288	147	< .001	.76
DAS-4		9.7	1.3	9.8	1.1	-1.440	133	n.s.	–
DASS Depression		3.6	3.5	2.9	3.3	4.021	142	< .001	.34
DASS Stress		6.5	4.1	5.3	3.9	4.122	131	< .001	.36
PPC		4.5	3.6	2.9	3.2	6.938	121	< .001	.63

*Notes*: *SDQ* Strengths and Difficulties Questionnaire, *ECBI* Eyberg Child Behavior Inventory, *GHQ* General Health Questionnaire, *PS* Parenting Scale, *PSOC* Parent Sense of Competency, *DAS-4* Dyadic Adjustment Scale, *DASS* Depression Anxiety Stress Scales, *PPC* Parent Problem Checklist, *n*.*s*. not significant.

† *p-*values corrected using Benjamini-Hochberg with false discovery rate set to 5%.

* For some analyses, n’s differ because of missing values, as can be seen in the *df* column.

For parents, significant differences after participation in Triple P group were found for GHQ-12, PS Laxness, PS Over-reactivity, PSOC, DASS Depression, DASS Stress, and PPC. No significant change was found for the DAS-4 scale. The results imply significant improvement in parent’s mental health, self-efficacy and self-reported parenting, but not parental relationship.

### Adoption

#### Setting level

During the second wave of implementation, 165 preschools, private and public, were eligible to participate in promoting and offering Triple P. Preschool principals were informed about the implementation in digital communications, such as emails and newsletters, as well as during common monthly meetings. Participation was optional, and decisions were made at preschool level. Preschools accepting to participate were used as recruitment bases for families, as well as providing Triple P practitioners with venues after day-care hours. Five private and six public preschools chose to opt out, hence no recruitment of parents took place at those venues, resulting in an adoption rate of 93.3%. This rate remained unchanged throughout the study period. To manage staff training, booking of venues, recruitment, planning and organisation of the overall implementation, two coordinators were recruited for the second wave of implementation. The coordinators were preschool teachers with Triple P training. They received basic leadership training via the municipality, and were also informed about implementation science from the university staff involved in the study. The Triple P program was financed and organised centrally and, hence, there was no extra cost for participating preschools compared with the ones opting out.

Analyses of Socioeconomic Structure Compensation index scores showed no significant differences between participating and non-participating preschools. Consequently, it was assumed that the participating preschools were representative of preschools in the municipality in terms of socio-economic status, educational level, and parents born outside Sweden.

#### Staff level

The practitioners in this study were preschool teachers, who self-selected to attend the Triple P training program. The Triple P training consisted of two days training at level 2 and 3, followed by three days of level 4 training for staff who had signed up for all the three levels. Following the training days, all trainees received supervision in sub-groups for 4–6 hours a week while holding their first seminars, counselling sessions, and groups. Trainees also received paid 13 study hours during this 6-week period. Further, a pre-accreditation session was offered in the middle of the post-training period. Accreditation days were scheduled 6–7 weeks after initial training. At these workshops, trainees were given the opportunity to demonstrate their competencies while receiving feedback and coaching on their performance. Trainees who did not successfully demonstrate their proficiency received further coaching from trainers and colleagues, and attended a later accreditation session. In order to become accredited practitioners, trainees also needed to complete a 30 question multiple-choice quiz before accreditation day, with at least an 80% score. Out of 25 trained practitioners, five never continued delivering the program after accreditation. The assessed staff adoption rate was therefore 80.8%.

The Triple P practitioners had monthly peer support-meetings, where they sat down, together with the coordinators, and addressed barriers or questions related to implementing the program. At these sessions, some practitioners who delivered seminars only expressed an unease to deliver groups due to too long time having passed since their training. Hence, booster sessions were offered for the these practitioners, tailored based on their expressed wants and needs in order to deliver Triple P group.

There was a change in recruitment of Triple P group practitioners among preschool staff during the course of the study. During the 2009–2013 study period, the Triple P training was open for all preschool teachers to attend. However, some practitioners were unable to deliver or delivered very few seminars and/or groups, due to difficulties in taking time off from other work-related duties, other assignments competing over the same resources of time and engagement, or lack of motivation. Hence, from 2014, the coordinators supplemented the recruitment process of new practitioners with an interview, assessing motivation, potential to work evenings and weekends, and personal engagement. Further, practitioners trained to deliver seminars were offered supplementary training to become Triple P group practitioners. This change in recruitment process was maintained throughout the second study period and has now become a routine practice.

### Implementation

#### Adaptations

As a result of discussions with professionals within psychology, pedagogy and with parents, some cultural adaptations of Triple P materials were made to better fit Swedish culture and views on parenting. The parenting technique *time-out* was adapted and renamed *wind-down* and *quiet time* technique to *pause*, with descriptions of the techniques putting more emphasis on regulating emotions and less on discipline. Further, the technique *planned ignoring* was reformulated, explicating the focus on child behaviours rather than child traits, i.e. not ignoring the *child* per se, but the behaviour. All changes were made in conversation with program developers and Triple P International Ltd.

To accommodate needs of parents unable to find child care for their children in connection with the Triple P group sessions, day carers were available on site, offering care and play in a child-friendly environment in close approximation to the parents. The day carers were supervised by early education professionals monthly to ensure a high quality care for the children. There were also refreshments offered to participants, both parents and children, during the sessions.

A delivery adaptation was made in 2017, as a consequence of interviewing previous participants. Some parents expressed difficulties attending the Triple P groups on weekday evenings. Hence, in addition to delivering groups on weekday evenings, weekend-groups were introduced, where all the content from the first four group sessions was fitted into two full days.

#### Parent recruitment

Group attendance and the number of groups delivered were lower than anticipated at first, which led to actions to increase both participation and delivery of the program. To increase outreach to different parents, flyers about Triple P were made available at preschools, within the child health services and at public libraries. The flyers for Triple P group were redesigned to appeal to mothers and fathers alike (see S2 Appendix). It was explicitly mentioned that the program discussed behavioural problems as well as emotional problems, as previous studies have showed that mothers are more likely to seek help for child behavioural problems and fathers for emotional problems [38]. They also contained relevant quotes from both mothers and fathers, implying that the program was targeted towards fathers as well, and that fathers were already attending the program. The front page image and text called attention to parenting not always being so easy, due to previous research on the greater potential of preventive versus promotive messaging in eliciting parental response to ads on parenting programs [44]. The average rate of fathers participating in Triple P group increased from 20.7% before the new flyers were introduced to 44.6% during 2017–2018.

#### Staff

Following the first study period (2009–2013), the need for coordinating and monitoring the implementation was apparent. Therefore, two of the trained preschool teachers were hired full-time as Triple P coordinators by the municipality. They continued delivering the program directly, but their main focus shifted towards implementation-related tasks. They planned for seminars and groups, organised the efforts to promote program participation, answered parents’ inquiries about the program, and communicated about the program to politicians and officials within the municipality. Further, five preschool teachers had 20% of their employment time dedicated to Triple P implementation, including planning, recruitment and delivery of the program. An additional six staff members were paid by the hour to deliver the program.

The coordinators took measures to include parents who did not have Swedish as their native language. Arabic is the second largest spoken language in Sweden, after Swedish. Thus, in an attempt to attract the large subgroup of Arabic-speaking parents, Triple P group in Arabic were introduced in 2017 and were since offered once per semester throughout the study period. Furthermore, parents not fluent in Swedish were offered to participate at any Triple P seminar with help from an interpreter, free of charge. In addition, seminars were offered in Arabic and English, albeit once per semester. Parents with hearing difficulties were offered sign language interpreters, free of charge.

The total yearly intervention costs were approximately €200,000. This included practitioner training; costs (salary, insurance, travel expenses) for coordinators, practitioners and day carers; Triple P material (e.g. workbooks) for attending parents; phones for practitioners; refreshments; and interpreter costs.

### Maintenance

#### Individual level

At 12 months after program participation, follow-up questionnaires indicate that several of the results from pre–post analyses were persistent (see Table 3 for a breakdown of the results from the 12 month follow-up analyses for child and parent outcomes). Of the SDQ total scores and subscales, improvements in Total score, Conduct Problems and Prosocial Behaviour remained significant. ECBI Intensity and Problem scores remained significantly improved at 12 months follow-up. For parents, improvements in parenting behaviours, self-efficacy, stress symptoms and inter-parental conflict remained significant.

**Table 3:** T-tests for Triple P group 12 month follow-up assessment.

		Pre		12 months					* *
*Child outcomes* (*n* = 48)*		Mean	*SD*	Mean	*SD*	*t*	df	*p* ^†^	*Cohen’s d*
SDQ Total		10.0	4.8	8.5	5.2	2.351	47	.039	.66
	SDQ Emotional Symptoms	1.4	1.5	1.4	1.7	-0.165	47	n.s.	–
	SDQ Conduct Problems	3.0	1.8	2.1	1.8	3.158	47	0.07	.53
	SDQ Hyperactivity	4.0	2.4	3.6	2.6	1.867	47	n.s.	–
	SDQ Peer Problems (*n* = 48)	1.5	1.8	1.3	1.4	0.829	47	n.s.	–
	SDQ Prosocial (*n* = 48)	6.8	2.0	7.5	2.1	-2.324	47	.039	.44
ECBI Intensity (*n* = 48)		120.2	28.7	106.0	22.4	4.354	47	< .001	.86
ECBI Problems (*n* = 45)		11.4	6.3	5.2	5.8	5.979	44	< .001	.93
*Parent outcomes* (*n* = 70)*									
GHQ-12 (*n* = 70)		11.6	5.3	10.9	5.4	1.194	69	n.s.	–
PS Laxness (*n* = 69)		3.1	0.7	2.9	0.7	6.096	68	.005	.49
PS Over-reactivity (*n* = 69)		3.3	0.8	2.9	0.8	9.107	68	< .001	.71
PSOC Efficacy (*n* = 70)		26.9	5.3	29.8	5.2	-9.787	69	< .001	.86
DAS-4 (*n* = 64)		9.7	1.3	9.7	1.1	-1.768	63	n.s.	–
DASS Depression (*n* = 68)		3.5	3.5	2.9	3.4	3.508	67	n.s.	–
DASS Stress (*n* = 65)		6.3	4.1	5.0	3.6	4.32	64	.002	.34
PPC (*n* = 59)		4.1	3.6	2.5	3.0	6.813	58	< .001	.78

*Notes*: *SDQ* Strengths and Difficulties Questionnaire, *ECBI* Eyberg Child Behavior Inventory, *GHQ* General Health Questionnaire, *PS* Parenting Scale, *PSOC* Parent Sense of Competency, *DAS-4* Dyadic Adjustment Scale, *DASS* Depression Anxiety Stress Scales, *PPC* Parent Problem Checklist, *n*.*s*. not significant.

† *p-*values corrected using Benjamini-Hochberg with false discovery rate set to 5%.

* For some analyses, n’s differ because of missing values, as can be seen in the *df* column.

#### Setting level

A signed agreement between the university and municipality that was followed up annually allowed for close collaboration between the two partners, focusing on continuous evaluation of the implementation process. During the entire implementation period, politicians and stakeholders within the municipality were informed about the implementation progress, including reports on attendance, demographic composition of attending parents, program evaluations by parents, costs, and preliminary pre–post results. The university researchers provided politicians and municipal stakeholders with annual reports about collaboration with the coordinators, covering reach, preliminary effectiveness, adaption, implementation progress, and maintenance-related planning.

In addition to yearly meetings with politicians and stakeholders, the coordinators arranged activities aimed toward the general public, including information days at malls and libraries, as well as themed one-day parenting forums where the public could meet coordinators, practitioners, researchers, politicians and Triple P participants.

Previous research has shown that a combination of facilitation strategies (a multi-faceted approach) is effective in maintaining program delivery [87]. A facilitator network was developed and maintained throughout the study period (and is ongoing) to support Triple P practitioners during training and delivery. In addition, parent advisors were engaged and invited to yearly network meetings, sharing their experiences. Some of these parents were also willing to talk to politicians, the media and support public opinion in favour of the program.

The Triple P program is still ongoing, more than ten years after initial planning and implementation and two years after the research project ended. The principles in the municipality’s policy for sustainable development, based on the United Nations’ sustainable development goals [88], stress learning perspectives on processes and actions towards changes in social sustainability [89], which resonates well with the continuous evaluation of the implementation process of the Triple P parenting program as a preventive universal intervention.

## Discussion

### Reach

Research suggests that parents who report higher levels of child behaviour problems are more likely to complete participation in a parenting program [90], but that different formats and intervention intensity are needed to reach as broadly as possible in terms of socioeconomic background, burden of problems, and needs [23, 91, 92]. Several efforts were made to attract parents with different socioeconomical and cultural backgrounds. This included availability of interpreters for non-Swedish speaking parents, and concentrated efforts to spread program information in low-income areas and areas with lower education levels. Program participation in group Triple P was increased among parents from non-Swedish speaking background and those with lower education after these efforts were put into practice. Parents with higher education were still over-represented in the study compared with the general population. However, since parents with lower education are less likely to fill in study questionnaires, participation in Triple P intervention might not have differed based on parental education [38].

Nevertheless, it is important to consider that previous research recommends awareness of increased inequalities as an unwanted side-effect of universally offered parenting interventions through, among other things, a skewed distribution of socioeconomic variables among recruited parents [40]. Further, as there is evidence that some (effective) public health interventions, such as smoking cessation programs, might increase inequalities due to different response to the intervention based on socioeconomic differences [93], it can be argued that parenting programs carry this potential risk as well. This topic was not assessed in the present study. However, in a systematic review and meta-analysis assessing such potential differences for the parenting program the Incredible Years, Gardner and colleagues found no such evidence [91].

The families participating in Triple P group had heightened scores on measures of child behaviour problems and poorer parental mental health compared with the general population. While Triple P groups were open for all parents to participate, this suggests that there might be a case of self-selection at play, in terms of parents and children with more difficulties attending to a higher degree. Further, a higher proportion of participating parents were seeking parenting support for boys. As studies suggest that boys express more regulation difficulties than girls during preschool-age [62, 94, 95], this uneven gender distribution might also indicate higher behaviour problems among the children in the Triple P groups. The Triple P system is designed as a stepped intervention, providing more intense parenting support at higher levels. Although we cannot know the actual scores of families in Triple P seminars, it could be argued that the sheer number of seminar participants suggests that parents might select intervention level based on needs. For future studies, this would be highly interesting to assess.

The number of individual counselling series were too few to be included in the study. This might be seen as a limitation since this level 3 of Triple P was not successfully implemented during the study period. However, reports from the Triple P coordinators from 2019 and onwards show a steady increase in the number of individual counselling sessions. This increase has continued in the wake of the COVID-19 pandemic as individual counselling is delivered via video calls, thus enabling participation in spite of restrictions on social gatherings.

Due to the lack of clear inclusion criteria and retrieving the number of children in the areas where Triple P was offered, it is difficult to find a valid denominator for assessing reach in relation to the municipal population. This can be seen as a study limitation.

### Effectiveness

The study design included no control group, which calls for cautiousness when interpreting the results of the outcome variables in terms of effects of Triple P. However, the statistically significant change in child and parent outcomes is in line with several RCT’s examining the effectiveness of Triple P [25, 26, 28]. While not being an RCT, the present study is instead representative of actual practice and thus provides valuable information on successes and challenges related to the implementation of parenting support in a real life setting. Displays of everyday life behaviour problems as measured by the ECBI suggest that the participating children benefit from Triple P group, and is an indication of the program’s usefulness.

In a meta-analysis of Triple P, Nowak and colleagues [28] concluded that there is a lack of measures of positive child behaviour in all analysed studies. Unfortunately, the study at hand is lacking positive health and behavioural measures as well, including only the prosocial subscale of the SDQ to assess positive child outcomes. It can be argued, that while many of the components of Triple P and other parenting programs are promotive, the outcome measures are prevention-focused. This could potentially be assessed further in future studies.

Parent mental health, parenting behaviour, and parent self-efficacy were significantly improved after program participation. While parents reported no significant improvement in general partner relationship quality, results indicate less conflict in their relationship regarding childrearing issues.

### Adoption

Both setting level and staff level adoption was considered successful, in regards to the number of participating preschools and their characteristics as measured with the Socioeconomic Structure Compensation index. At staff level, accreditation rate was 100%, which can be considered high compared with 82% in a previous population study of Triple P [32]. Further, the accredited practitioners continued delivering the program at a similar or higher rate than many previous Triple P studies report [29–32]. This success can be attributed to (1) the voluntary nature of training (it was individual preschool teachers themselves and not their managers who could decide whether or not to express interest in becoming Triple P practitioners), (2) the selection process (interested preschool teachers were carefully selected based on their motivation, engagement and availability), and finally (3) regular paid peer-supervision meetings for all Triple P practitioners. However, while the number of active practitioners was satisfactory, there was a lack of information on the fidelity to the Triple P protocol at staff level. This could be seen as a study limitation, as fidelity measures would expand the understanding of the implementation of Triple P in this particular setting.

### Implementation

Insights from implementation science were used in a number of ways to support implementation, fidelity, and adequate program delivery. The importance of monitoring implementation has for example been reported in a review by Durlak and DuPre [96], and was addressed in the present study by the inclusion of implementation coordinators. Triple P coordinators set up regular peer supervision meetings that has previously been identified as an important facilitator of program use among Triple P practitioners [30, 97]. Audit and feedback using the program evaluation questionnaires were used to monitor delivery quality, but also to provide feedback to practitioners. Delivery was monitored along with costs and presented annually to municipal politicians and decision-makers to maintain financing and organisational support for the program.

There is a need to adapt program content when implementing research-supported interventions, for improving the actual intervention outcomes as well as the implementation outcomes [98]. In fact, adapting, rather than simply adopting the interventions has been shown to increase their effectiveness [99]. In the present study, the Triple P parenting techniques were adapted to a Swedish context, weekend-groups were made available, and child care and refreshments were offered at seminars and groups. The purpose of the adaptations was to make the program relevant within the Swedish context and to enable parents living far from the sites where groups were offered to attend, as well as families in need of child care while attending Triple P.

### Maintenance

Both program delivery and observed reduction of some measures of child behaviour problems and improved parent mental health were maintained over time. For child outcomes, changes on the SDQ Conduct Problems subscale and both ECBI scales remained significant at 12 months follow-up. Thus, there seems to be a general stable improvement in behaviour problems for the children whose parents attended Triple P group. Sample size at follow-up was smaller than post intervention, which may have impact on significance, as several of the outcome mean values at post and follow-up are similar. The significant improvements in parent outcomes were maintained for parenting behaviour, parent self-efficacy, parental stress, and conflicts between parents. This implies that many changes detected at post-intervention analyses were stable over time. The positive changes in the DASS Depression and GHQ scales, which measure depressive symptoms, did not stay significant at 12 months follow-up. This could be due to a reversion of depressive symptoms, implying that these changes were not stable over time. However, similar to child outcome assessment, the sample size for the 12 month follow-up analyses was rather small, and mean values at post and 12 months follow up are similar for the DASS Depression scale but not for the GHQ. Analyses using a larger sample would be preferable to address this question. Further, due to constraints related to sample size, we were not able to assess long-term effects on subgroup level. Thus, we cannot make claims of robustness across subgroups, either at post attendance or over time.

Viewing the study results in the light of Weeks’ [51] identified factors for successfully implementing research-supported interventions, several of the key factors were addressed. The implementation process involved outside-organisation factors such as collaboration between researchers, politicians and Triple P coordinators, as well as inside-organisation factors including supervision and leadership engagement. As mentioned in the introduction, staff “buy-in” was assessed and reported prior to the current study [48] and indicated that the preschool staff saw positive aspects of the program and usefulness within their own professional work.

## Conclusion

The present study described the successful implementation of the multilevel Triple P parenting program using preschools as an arena in a Swedish municipality. The reach, effectiveness, adoption, implementation and maintenance of the program were all satisfactory and demonstrated the suitability of delivering evidence-based parenting support using preschools as an arena and preschool teachers as program facilitators. Given the high prevalence of behaviour problems in preschool children, the corresponding parental mental health problems [100] and the negative long-term outcomes of sustained behaviour problems, this study should provide guidance for decision-makers regarding the implementation of parenting programs using the preschool system. Although no news to those well acquainted with the implementation literature, this study once again shows that careful implementation support is necessary for success.

## Supporting information

S1 Appendix Triple P seminars evaluation questionnaire. (PDF)

S2 Appendix Triple P group flyer. (PDF)
