# Supplementary material for: Successful implementation of parenting support at preschool: An evaluation of Triple P in Sweden
Source: PLoS One. 2022 Apr 13;17(4):e0265589. doi: 10.1371/journal.pone.0265589 (PMC9007376; doi:10.1371/journal.pone.0265589)
Supplement: S1 Appendix — (PDF) [file pone.0265589.s001.pdf]

Frågor om seminariet
Questions regarding the seminar

	Dålig/t<br>*Poor*						Utmärkt<br>*Excellent*
Hur skattar du kvaliteten på presentationen?<br>*How would you rate the quality of the seminar?*	1	2	3	4	5	6	7
Hur skattar du föreläsningens innehåll?<br>*How would you rate the content of the seminar?*	1	2	3	4	5	6	7

	Nej definitivt inte<br>*No, definitely not*						Ja Definitivt<br>*Yes, definitely*
Gav föreläsningen tillräckligt utrymme för frågor?<br>*Did the seminar leave enough room for questions?*	1	2	3	4	5	6	7
Använde utövaren tydliga exempel för att illustrera föräldraskapsbekymmer?<br>*Did the practitioner use clear examples to illustrate parenting issues?*	1	2	3	4	5	6	7
Tänker du använda något av de råd om föräldraskap du fått?<br>*Will you use some of the advice that you received?*	1	2	3	4	5	6	7
Skulle du rekommendera någon annan att gå på den här föreläsningen?<br>*Would you recommend someone else to attend this seminar?*	1	2	3	4	5	6	7
Kan du tänka dig att delta på fler föreläsningar?<br>*Would you consider attending more seminars?*	1	2	3	4	5	6	7
Har du fått tillräcklig kunskap och information för att kunna tillämpa de råd om föräldraskap som du fått?<br>*Have you gained enough knowledge and information to implement the parenting advice that you have received?*	1	2	3	4	5	6	7
