# Supplementary material for: Successful implementation of parenting support at preschool: An evaluation of Triple P in Sweden
Source: PLoS One. 2022 Apr 13;17(4):e0265589. doi: 10.1371/journal.pone.0265589 (PMC9007376; doi:10.1371/journal.pone.0265589)
Supplement: S2 Appendix — (PDF) [file pone.0265589.s002.pdf]

[removed logo]

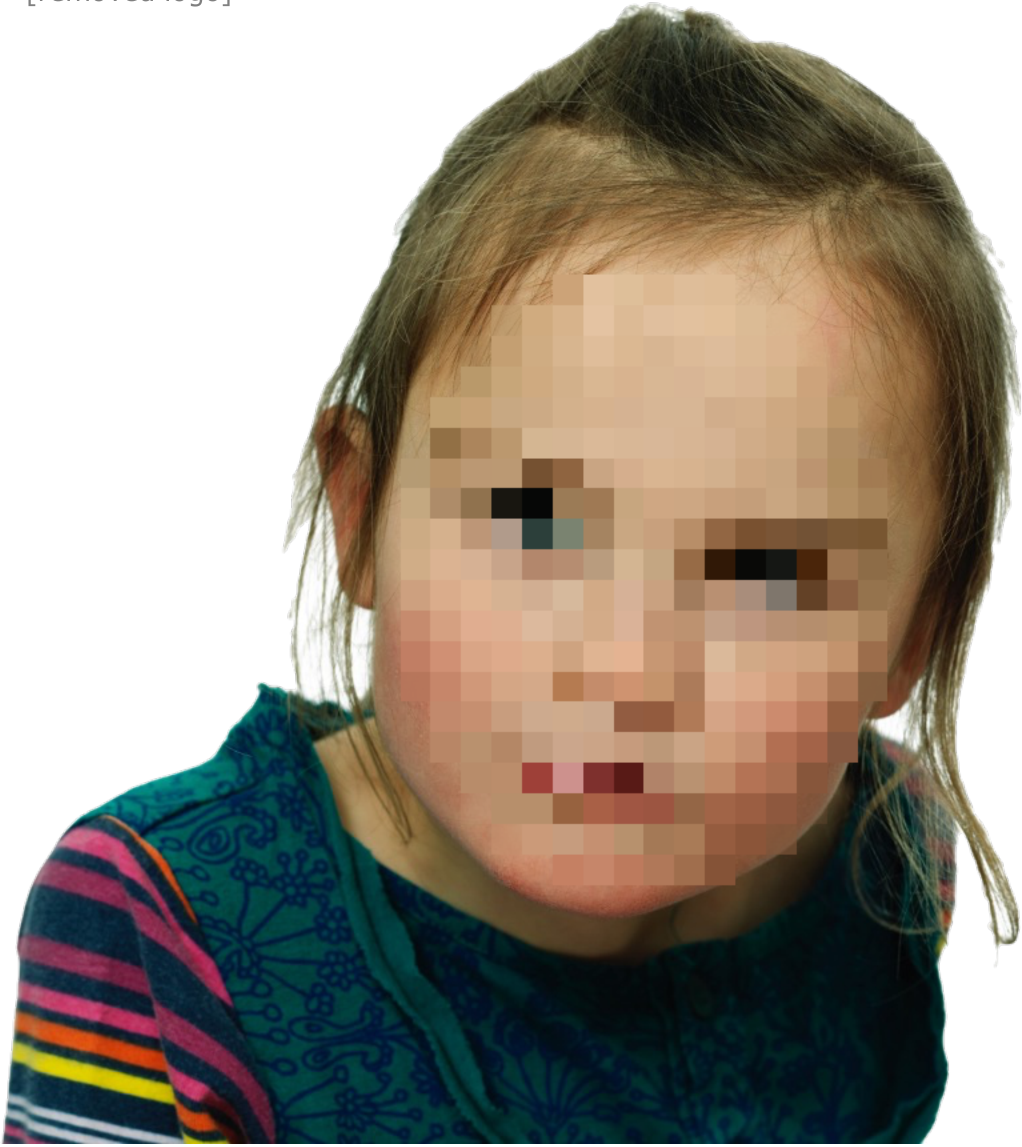

It is not always easy to be a parent

– with Triple P you can strengthen your parenting skills

[removed logos]

Free parenting support

All parents of 0-5 years old children in Uppsala municipality are offered parenting support. Triple P – Positive Parenting Program – was developed at The University of Queensland in Australia, and has been available in Sweden since 2008.

Do you find yourself in difficult and challenging family situations several times a week?

Would you like to meet other parents and get tools that you can use to feel more confident in your parenting role?

Then **Group Triple P** may be for you.

Triple P is based on over 35 years of research that shows parents who have received Triple P have better parenting skills and feel more confident in their parenting role. Moreover, their children have fewer behavioural and emotional problems, do better at school and make friends more easily.

Group Triple P includes 8 sessions

Four group sessions

Two hours each, once per week for four weeks

Suggestions about what you can do to
- create a calm and positive environment at home
- strengthen the relationship with your child

Reflection about
- how your family works
- what you may like to change as a parent

Learning about how you can
- better manage your child's problem behaviours, such as nagging and tantrums
- strengthen your child's self-esteem
- teach your child to recognise and accept different feelings
- take care of yourself as a parent

You receive support in trying out new tools and strategies in your everyday life at home.

Three phone calls

15-30 minutes each, once per week for three weeks

The facilitator calls you, offering you support in using the strategies you have discussed during the group sessions.

A final group session

Two hours, once

You assess your progress over the course of the program and discuss how you can maintain the positive changes you have made.

We offer light refreshments during the group sessions. Free onsite childcare is available on request. Please let us know in advance if you need an interpreter.

Comments from parents

Any parent can take this. You can have good kids and still struggle with something.

Father and mother of two

My wife and I gained an incredible amount of practical information given in a friendly non-invasive environment. The advice was presented in an easy-to-understand format.

Father of two

I have learnt a lot about how to manage my child's tantrums.

Mother of two

With the help of Triple P, there is not nearly as many conflicts, and when there is a conflict, often we can manage it easily. We feel better, act calmer and the whole family have more fun together

Father and mother of one

Interested?

Read more at: ***http://triplep.uppsala.se/*** or contact us if you have questions.

uppsala.se

E-mail:
foraldravagledning@uppsala.se

facebook

Phone:
Anna Hanning, Triple P co-ordinator, 018-727 56 51
Agneta Nyberg, Triple P co-ordinator, 018-727 56 52

New groups start in the beginning of 2017.

Places are limited.
Register your interest as soon as possible!
